# Genome-Wide Analysis of the *RNase* T2 Family and Identification of Interacting Proteins of Four *ClS-RNase* Genes in ‘XiangShui’ Lemon

**DOI:** 10.3390/ijms231810431

**Published:** 2022-09-09

**Authors:** Yu-Ze Li, Jia-Wei Zhu, Wei Lin, Mo-Ying Lan, Cong Luo, Li-Ming Xia, Yi-Li Zhang, Rong-Zhen Liang, Wang-Li Hu, Gui-Xiang Huang, Xin-Hua He

**Affiliations:** 1 State Key Laboratory for Conservation and Utilization of Subtropical Agro-Bioresources, College of Agriculture, Guangxi University, 100 East Daxue Road, Nanning 530004, China; 2 Institute of Subtropical Agriculture, Fujian Academy of Agricultural Sciences, Zhangzhou 363005, China

**Keywords:** ‘XiangShui’ lemon, S-RNase, self-incompatibility, expression analysis, functional analysis

## Abstract

S-RNase plays vital roles in the process of self-incompatibility (SI) in *Rutaceae* plants. Data have shown that the rejection phenomenon during self-pollination is due to the degradation of pollen tube RNA by S-RNase. The cytoskeleton microfilaments of pollen tubes are destroyed, and other components cannot extend downwards from the stigma and, ultimately, cannot reach the ovary to complete fertilisation. In this study, four S-RNase gene sequences were identified from the ‘XiangShui’ lemon genome and ubiquitome. Sequence analysis revealed that the conserved RNase T2 domains within S-RNases in ‘XiangShui’ lemon are the same as those within other species. Expression pattern analysis revealed that *S_3_-RNase* and *S_4_-RNase* are specifically expressed in the pistils, and spatiotemporal expression analysis showed that the S_3_-RNase expression levels in the stigmas, styles and ovaries were significantly higher after self-pollination than after cross-pollination. Subcellular localisation analysis showed that the S_1_-RNase, S_2_-RNase, S_3_-RNase and S_4_-RNase were found to be expressed in the nucleus according to laser confocal microscopy. In addition, yeast two-hybrid (Y2H) assays showed that S_3_-RNase interacted with F-box, Bifunctional fucokinase/fucose pyrophosphorylase (FKGP), aspartic proteinase A1, RRP46, pectinesterase/pectinesterase inhibitor 51 (PME51), phospholipid:diacylglycerol acyltransferase 1 (PDAT1), gibberellin receptor GID1B, GDT1-like protein 4, putative invertase inhibitor, tRNA ligase, PAP15, PAE8, TIM14-2, PGIP1 and p24beta2. Moreover, S_3_-RNase interacted with TOPP4. Therefore, S_3_-RNase may play an important role in the SI of ‘XiangShui’ lemon.

## 1. Introduction

Self-incompatibility (SI), in which pistils and stamens develop properly but cannot self-pollinate, fertilise and produce fruit, is a common phenomenon in plant genetic processes. With SI, inbreeding is effectively avoided, and the probability of extinction is greatly reduced. At present, most studies on SI have focused on the *Cruciferae*, *Solanaceae*, *Rosaceae*, *Plantaginaceae* and *Papaveraceae* plant species [1]. Studies on SI in *Rutaceae* plants are quite rare.

Many studies have reported that SKP1 physically interacted with the F-box (SLF) and Cullin1 proteins. SKP1, as a bridge protein, promotes the connection of SLF to Cullin1 to form the SCF (F-box-SKP1-Cullin1) complex, which mediated the degradation of S-RNase proteins by the ubiquitination pathway. S-RNases, which are key genes mainly involved in SI, play important roles in this process. Many studies have reported that S-RNase-style proteins enter pollen tubes through transmembrane transport, after which the nuclear DNA of pollen tubes is degraded, the RNA of pollen tubes is degraded and calcium influx increases. In addition, the mitochondrial membrane potential changes and the membrane structure is destroyed; afterwards, the microfilament cytoskeleton is depolymerised, causing programmed cell death (PCD) in the pollen tube. In *Antirrhinum hispanicum*, an S-RNase gene was shown to interact with the *AhSLF-S_2_* gene [2]; in apple (*Malus domestica*), *MdSFBB1* interacts with an S-RNase [3]. Two S-RNase genes have been cloned from *Pyrus bretschneideri*, and it was proven that PbS_21_-RNase interacts with PbSLF6-S_21_ and PbSLF_3_-S_34_ [4].

In *Rosaceae*, non-S determinants interacting with S-RNases have been successively discovered. A major breakthrough had been achieved in studying the mechanism through which the toxic effect of S-RNase proteins is regulated under combined activity. S-RNases interact directly with the pollen tube microfilament skeleton protein PbrAct1 in pear (*Pyrus bretschneideri*), resulting in the actin cytoskeleton depolymerizing the self-incompatible pollen tube [5]. By screening pollen factors that could interact with S-RNases in apple, researchers found that the MdABCF transport protein regulates the depolymerisation of the microfilament cytoskeleton [6]. An actin-binding protein, MdMVG, was identified in apple that interacts with S-RNases to reduce self-pollen tube growth through the inhibition of its F-actin-severing activity [7]. MdPPa can interact with S-RNase, inhibiting the sPPase of the protein by changing its conformation and elevating the levels of inorganic pyrophosphate (PPi). This may lead to tRNA aminoacylation being inhibited, protein synthesis being blocked and the growth of pollen tubes being inhibited [8]. MdCaMBP can interact with S-RNases, after which the calcium gradient is affected indirectly, and the growth of the pollen tube is affected [9,10].

However, in the *Rutaceae* family, there are few studies on the non-S determinant of the interaction of S-RNases, key genes that drive SI. ‘XiangShui’ lemon belongs to the *Citrus L.* genus of the *Rutaceae* family and has the excellent SI seedless characteristic. Therefore, related genes that may interact with S-RNases in ‘XiangShui’ lemon were screened through a yeast two-hybrid (Y2H) experiment. To build an SI system that is different from the traditional ubiquitin degradation pathway of the SCF complex model, we searched for non-S determinants that may participate in the SI of ‘XiangShui’ lemon. Two ClS-RNase genes specific to pistils and one ClF-box gene specific to pollen have been identified in ‘XiangShui’ lemon [11].

In this study, eight RNase T2 genes were identified and analysed on the basis of the genomic and ubiquitin-modified-omic data [12] of ‘XiangShui’ lemon. Through a Y2H experiment, the putative regulatory network of S_3_-RNase was preliminarily proposed, which laid a foundation for future studies of the SI mechanism of ‘XiangShui’ lemon.

## 2. Results

### 2.1. Identification of S-RNase Genes in ‘XiangShui’ Lemon

Eight RNase T2 genes were identified from the genomic and ubiquitin-modified-omic data of ‘XiangShui’ lemon. The eight *RNase* T2 genes were named S_1_-RNase–S_8_-RNase. Among them, S_3_-RNase was present within the genomic and in the ubiquitin-modified-omic datasets. All the analysed proteins had an RNase T2 conserved domain. Details of the S-RNase proteins are listed in Table 1. The protein lengths ranged from 89 to 278 aa, the molecular weights ranged from 10.07 to 31.47 kDa, and the isoelectric points increased from 4.93 to 10.60.

**Figure 1:**
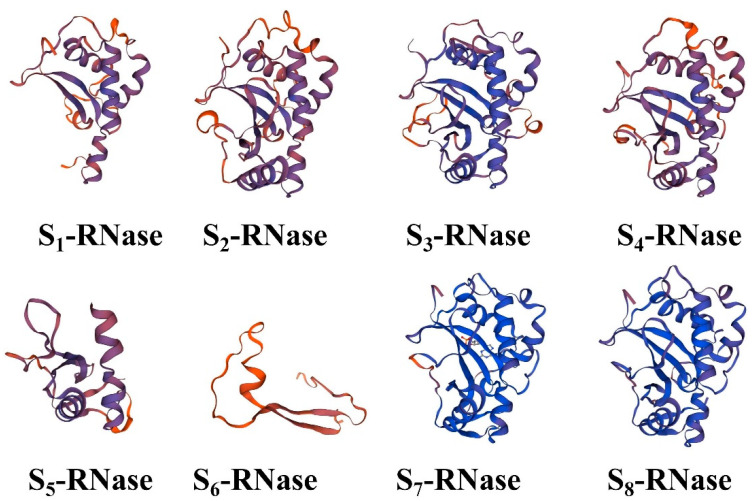
Three-dimensional structures of S-RNase proteins.

SOPMA software analysis showed that the secondary structure of lemon S-RNase proteins usually consisted of four components, namely, α-helices, β-turns, random coils and extended strands, as shown in Supplementary Table S2. These findings indicated that the similarity in the secondary structure of the same subregions of S-RNase proteins may form similar higher-order structures to perform similar functions. The proportion of random coils was the highest (51.49~38.78%), which was often affected by side-chain interactions and is closely related to the composition of the active sites in the proteins. The next highest proportion was that of α-helices (42.09~18.18%), followed by the proportion of extended strands (12.24~3.24%), while the proportion of β-turns was the lowest (11.91~3.24%). The tertiary structure of a protein was based on its secondary structure, with the protein curling and folding into a specific conformation with the help of various secondary bonds. Therefore, the online software SWISS-MODEL was used for homology modelling of the eight S-RNase proteins, and the majority of them were found to have homologous three-dimensional conformations (Figure 1).

### 2.2. Chromosomal Locations

**Figure 2:**
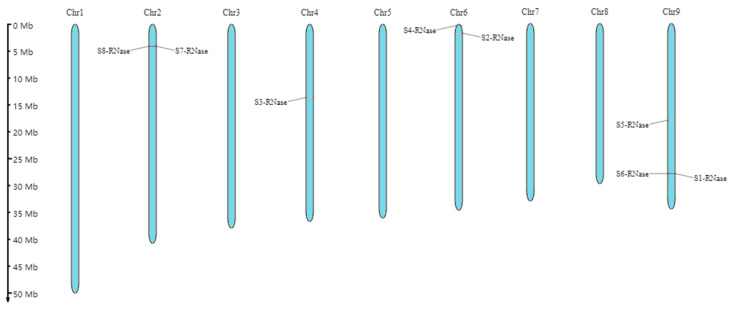
Chromosomal locations of eight S-RNases on the ‘XiangShui’ lemon chromosomes. The top of each scaffold shows the chromosome numbers. The chromosome sizes are shown with the vertical scale.

As shown in Figure 2, the S_8_-RNase and S_7_-RNase genes were distributed on chromosome 2, the S_3_-RNase gene was distributed on chromosome 4, the S_4_-RNase and S_2_-RNase genes were distributed on chromosome 6, and the S_5_-RNase, S_6_-RNase and S_1_-RNase genes were distributed on chromosome 9 (Figure 2).

### 2.3. Phylogenetic Analysis of S-RNase Proteins

**Figure 3:**
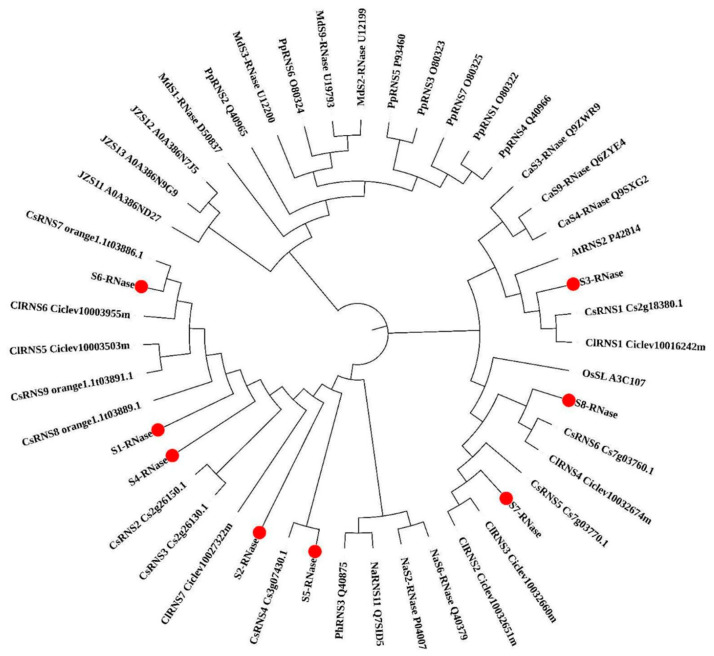
Phylogenetic analysis of S-RNase proteins in ‘XiangShui’ lemon, Arabidopsis, apple, pear, sweet orange and Citrus clementina. The phylogenetic tree included eight S-RNase (*Citrus × limon ‘Rosso’*), nine CsRNS (*Citrus sinensis*), seven ClRNS (*Citrus clementina*), one PhRNS3 (*Petunia hybrida*), seven PpRNS (*Pyrus pyrifolia*), one AtRNS (*Arabidopsis thaliana*), four MdS-RNase (*Malus domestica*), one OsSL (*Oryza sativa subsp*), three NaS-RNase (*Nicotiana alata*), three CaS-RNase (*Prunus avium*), and four JZS (*Ziziphus jujuba*).

The eight RNase T2 genes were clustered together with the Citrus sinensis, Citrus clementina and S-RNase genes identified in other species. Phylogenetic analysis showed that the eight S-RNase genes of the RNase T2 family cluster separately and cluster closest to the Citrus sinensis and Citrus clementina genes (Figure 3).

### 2.4. Analysis of S-RNase Gene Family Promoter Cis-Acting Elements

**Figure 4:**
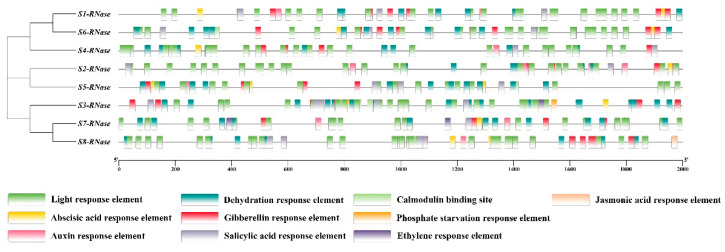
Cis-acting elements upstream of auxin response factor genes in ‘XiangShui’ lemon. The presence of cis-acting elements in a 2000 bp sequence upstream of the ATG start codons was assessed using PlantCARE and PLACE. The horizontal scale indicates the promoter length.

To further understand the expression regulation characteristics of the RNase T2 gene family in ‘XiangShui’ lemon, the cis-acting elements in the promoter were analysed in the 2000 bp promoter region upstream of the eight RNase T2 genes. The results showed that the eight RNase T2 genes contained multiple light-response elements, abscisic acid (ABA) response elements, auxin (IAA) response elements, dehydration responsive elements, gibberellin (GA) response elements and salicylic acid (SA) response elements. Calmodulin-binding sites are present only in S_3_-RNase, S_5_-RNase and S_6_-RNase, and ethylene response elements are present only in S_3_-RNase and S_7_-RNase. Jasmonic acid response elements are present only in S_8_-RNase, and phosphate formation response elements are present only in S_3_-RNase (Figure 4).

### 2.5. Analysis of the Gene Structure and RNase T2 Conserved Domain of S-RNases

**Figure 5:**
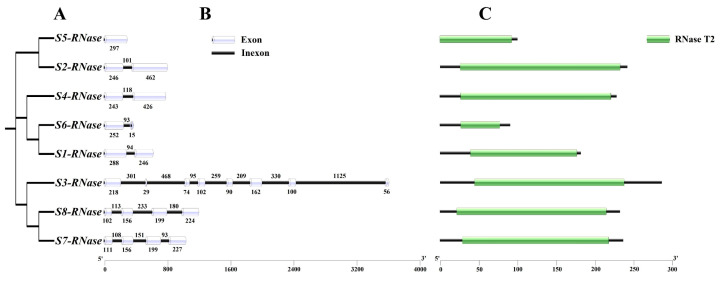
Phylogenetic relationships, structure and conserved domains of the S-RNase genes. (**A**) Construction of a rootless neighbour-joining phylogenetic tree comprising eight lemon S-RNase protein sequences. (**B**) Exon/intron structure of S-RNase genes. Exons are represented by the white cylinders, and the black lines represent introns. The scale at the bottom is in base pairs (bp). (**C**) Distribution of the conserved RNase T2 family domain in S-RNases.

The S-RNase gene structure showed that S_5_-RNase has no introns, S_2_-RNase, S_4_-RNase, S_6_-RNase and S_1_-RNase each have one intron, S_3_-RNase has seven introns and S_8_-RNase and S_7_-RNase have three introns. The eight S-RNases all have an RNase T2 conserved domain (Figure 5).

### 2.6. S-RNase Gene Expression Pattern Analysis

**Figure 6:**
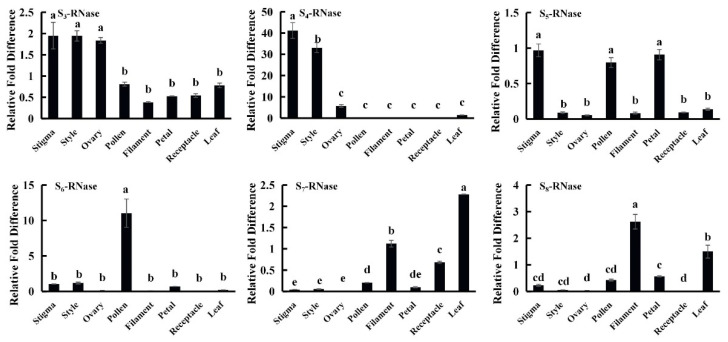
Transcription profiles of S-RNase genes in the stigmas, styles, ovaries, pollen, filaments, petals, receptacles and leaves. The relative expression level was calculated using the 2^−^^ΔΔ^^CT^ method. The values are the means ± SD (*n* = 3). The different lowercase letters represent significant differences at α = 0.05.

The expression pattern analysis of S-RNase genes in tissue revealed that S_3_-RNase and S_4_-RNase genes were specifically expressed in the pistils. Previous expression pattern analysis found that S_1_-RNase and S_2_-RNase were specifically expressed in the pistils [13], S_5_-RNase was highly expressed in the pollen, stigmas, and petals; S_6_-RNase was specifically expressed in the pollen; S_7_-RNase was highly expressed in the leaves; and S_8_-RNase was highly expressed in the filaments (Figure 6).

It has been reported that the S-alleles gene whose product has S-RNase activity is specifically expressed in the pistils. The expression patterns of S_1_-RNase, S_2_-RNase, S_3_-RNase and S_4_-RNase in ‘XiangShui’ lemon yielded similar results. Therefore, these four genes will be studied in the future.

**Figure 7:**
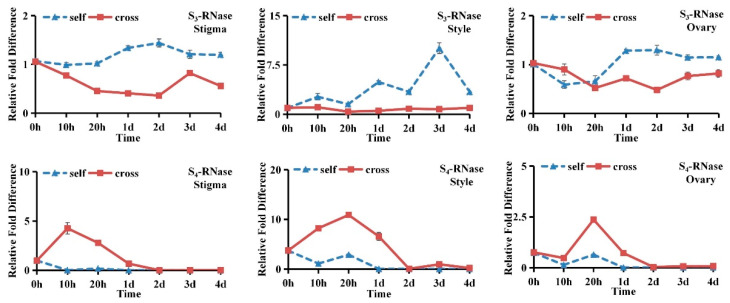
The transcription profiles of S-RNase genes in the Stigma, Style, Ovary, from 0 h to 4 d.

The spatiotemporal expression patterns of the S_3_-RNase and S_4_-RNase genes in ‘XiangShui’ lemon were analysed. The expression of the S_3_-RNase gene in stigmas, styles and ovaries began to increase significantly 20 h after self-pollination and peaked from 2 d to 3 d. The expression in the style was approximately ten times that after cross-pollination, and approximately three times that in the stigma and ovary. In previous studies, the expression pattern of the S_1_-RNase gene was found to be similar to that of the S_3_-RNase gene [11]. The spatiotemporal expression pattern of the S_4_-RNase gene in stigmas, styles and ovaries was not consistent with SI. Therefore, S_1_-RNase and S_3_-RNase likely participate in SI (Figure 7).

### 2.7. Subcellular Localisation

**Figure 8:**
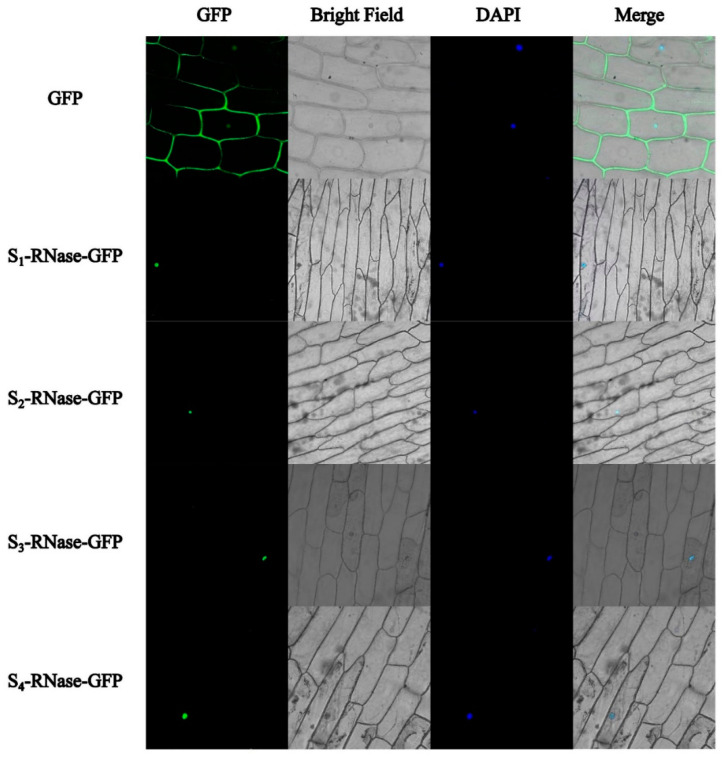
Subcellular localisation of four S-RNase proteins in Allium cepa L. GFP: Signalling of a GFP-fused protein by fluorescence microscopy. Merged: Superimposed GFP and bright-field images. (Bar = 100 μm).

The constructed S_1_-RNase-GFP, S_2_-RNase-GFP, S_3_-RNase-GFP and S_4_-RNase-GFP fusion expression vectors were transiently expressed in onion epidermal cells by the Agrobacterium-mediated method. Under a fluorescence confocal microscope, it was determined that the four proteins were localised in the nucleus (Figure 8).

### 2.8. Y2H Screening Assays

**Figure 9:**
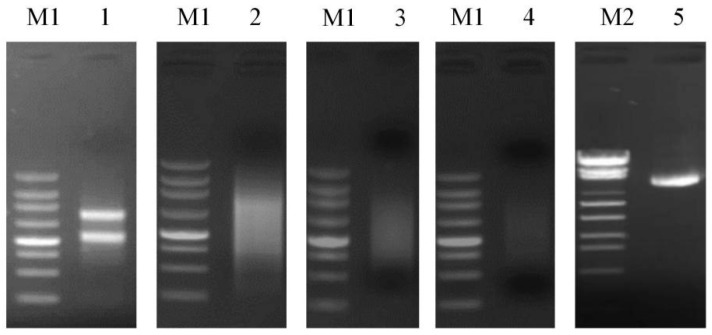
M1: 250 bp DNA Ladder (TaKaRa Code No.3424A). M2: Lambda EcoT14 I digest (TaKaRa Code No.3401). 1: Total RNA. 2: Synthetic cDNA. 3: Normalised cDNA. 4: cDNA after short segment removal. 5: Amplified plasmid.

Total RNA was extracted from mixed samples of ‘XiangShui’ lemon flower organ tissues. Afterwards, 300 ng of total RNA was used for 1.5% agarose gel electrophoresis, and the quality of the RNA was determined. The results are shown in Figure 9 and were used in subsequent experiments. The total RNA was reverse transcribed to cDNA. Five microlitres of cDNA was subjected to 1.5% agarose gel electrophoresis to determine the quality of the synthesised cDNA. The results are shown in Figure 9. The purified cDNA was then normalised. A total of 30 µg of cDNA was taken from a 1.5% agarose gel after electrophoresis, and the results are shown in Figure 9. After purification, the short cDNA fragment was removed and dissolved in dH2O. One microlitre of cDNA was subjected to 1.5% agarose gel electrophoresis, and the results are shown in Figure 9. The cDNA was diffused, and the size distribution ranged from 500–4000 bp. The quality was good, indicating that the cDNA could be used for library construction. The library capacity of the purified cDNA primary library was determined, and the library capacity was calculated to be approximately 5.0 × 10^6^ CFU. Forty-eight clones were randomly selected for PCR identification. The average length was more than 1 kbp, and the recombination rate was approximately 96%. The primary library was amplified, and the library plasmids were extracted and subjected to 1.5% agarose gel electrophoresis. The results are shown in Figure 9.

**Figure 10:**
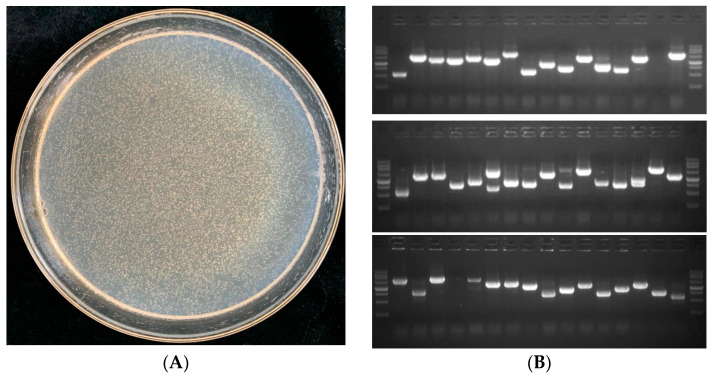
(**A**) Identification of the Y2H library. (**B**) Detection of cDNA inserts in the library.

The library plasmid was transformed into Y187 competent cells, diluted 100 times and coated onto a -Leu plate. The transformation efficiency was calculated according to the following equation: ‘Transformation efficiency = (Number of single colonies × Total volume of cell suspension, mL × Dilution ratio)/(Bacterial solution volume of coated plate, mL × Total DNA, μg)’. The library capacity was 3.5 × 10^7^ CFU/μg, and the requirements for use in subsequent experiments were met(Figure 10).

**Figure 11:**
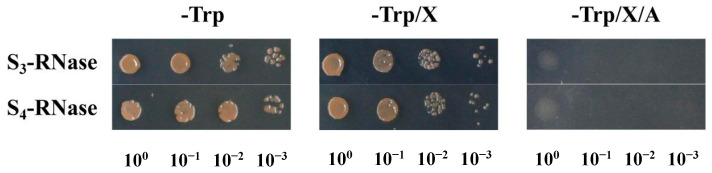
Transcriptional activation activity of S_3_-RNase and S_4_-RNase.

To continue to study the functions of the four S-RNases, the interacting proteins of S-RNases were screened by Y2H assays. The full-length open reading frame (ORF) was inserted in the pGBKT7 bait vector and transformed into Y2H gold competent cells. The results of self-activation showed that normally growing yeast cells transformed with S-RNase on SD/-Trp and SD/-Trp/X-α-gal media are pink, and these cells do not grow on SD/-Trp/X-α-gal/AbA media (Figure 11). It was proven that the four S-RNases did not exhibit self-activation; thus, subsequent experiments were performed.

**Figure 12:**
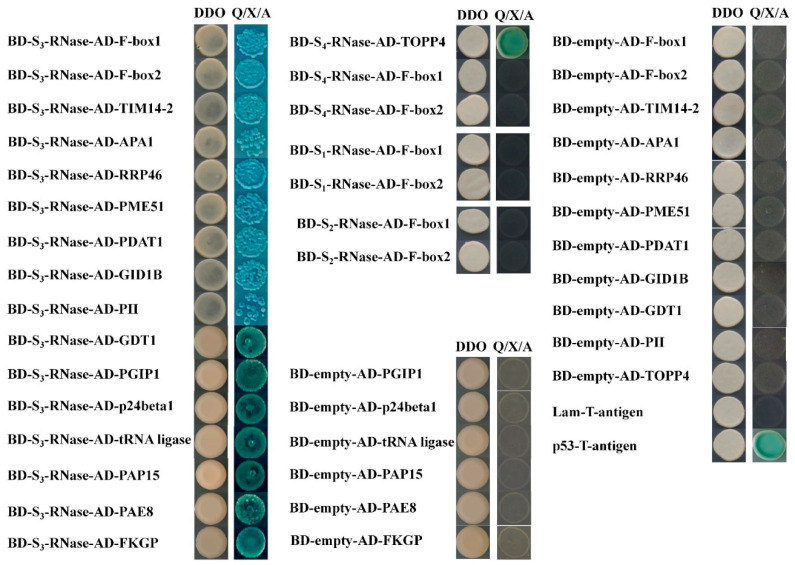
Point-to-point verification of S-RNase with candidate proteins.

Bait yeast was mixed with the library components and coated on QDO(SD/-Leu/-Trp/-His/-Ade) media. The colonies that turned blue on the plate were identified via PCR and were sent for sequencing confirmation. The results displayed after duplicate clones were excluded, and positive clones that interacted with S_1_-RNase and S_2_-RNase were not screened. A total of 41 blue monoclonal colonies were obtained via S_3_-RNase, and a total of 12 blue monoclonal colonies were obtained via S_4_-RNase. Fifteen SI-related proteins were selected for point-to-point assays. The results showed that S_3_-RNase interacted with F-box1, F-box2, mitochondrial import inner membrane translocase subunit TIM14-2 (TIM14-2), aspartic proteinase A1 (APA1), exosome complex exonuclease RRP46 homolog (RRP46), pectinesterase/pectinesterase inhibitor 51 (PME51), phospholipid:diacylglycerol acyltransferase 1 (PDAT1), gibberellin receptor GID1B, GDT1-like protein 4, putative invertase inhibitor (PII), Polygalacturonase-inhibiting protein 1 (PGIP1), transmembrane emp24 domain-containing protein p24beta2-like (p24beta2), Serine-tRNA ligase, purple acid phosphatase 15 (PAP15), pectin acetylesterase 8 (PAE8) and bifunctional fucokinase/fucose pyrophosphorylase (FKGP). Additionally, S_4_-RNase interacted with TOPP4 (Figure 12).

### 2.9. Tissue Expression Analysis of Interaction Candidate Genes

**Figure 13:**
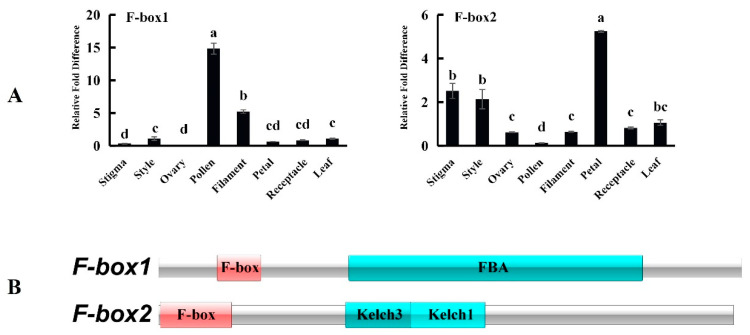
(**A**)Tissue expression patterns of F-box1 and F-box2 candidate proteins. (**B**) Predicted functional domains of F-box1 and F-box2. The relative expression level was calculated using the 2^−^^ΔΔ^^CT^ method. The values are the means ± SDs (*n* = 3). The different lowercase letters represent significant differences at 0.05.

The two screened F-box proteins are components of the SCF complex. NCBI BLAST queries revealed that the similarity between the F-box1 protein and Citrus sinensis F-box protein At5g03970-like (XM_006466714.3) is 98.98%, and the similarity between F-box2 and Citrus clementina F-box/kelch-repeat protein At1g15670 (XM_006427024.2) is 97.56%. Many studies have shown that the SCF complex can ubiquitinate the marker S-RNase and degrade S-RNase through the 26S proteasome. Studies have shown that the F-box involved in SI is specifically expressed in pollen, and most of it has an FBA domain at the C-terminal. Therefore, we analysed its tissue expression pattern and conserved domain. The results showed that F-box1 was specifically expressed in the pollen and had an F-box domain at the N-terminus and an FBA domain at the C-terminus. F-box2 has an F-box domain at the N-terminus and two Kelch domains at the C-terminus (Figure 13).

### 2.10. Bimolecular Fluorescence Complementation (BiFC) Experiments for Verification of the Interactions between S-RNases and F-box Proteins

**Figure 14:**
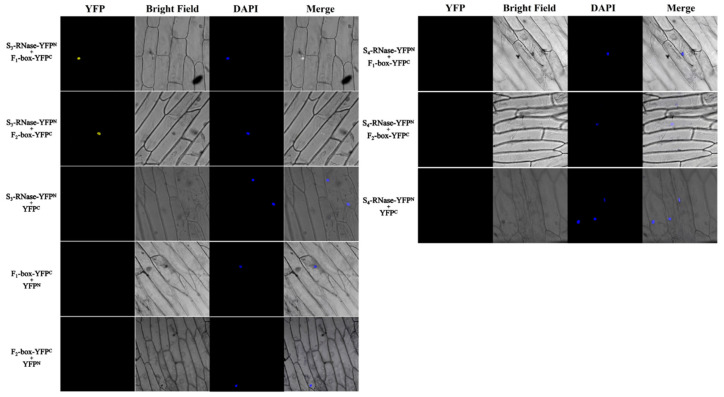
BiFC assays of S-RNases with candidate proteins. BiFC assays of S-RNases and candidate proteins with empty vectors. YFP: Signalling of a YFP-fused protein via fluorescence microscopy; DAPI: staining showing the cell nucleus; Merged: superimposed YFP, DAPI, and bright-field images. Bars = 100 µm.

The interaction between S_3_-RNase and two F-box proteins was verified by onion BiFC experiments. BiFC vectors puc-sPYNE-S_3_-RNase, puc-sPYNE-S_4_-RNase, puc-sPYCE-F-box1, and puc-sPYCE-F-box2 were subsequently constructed and tested. The results are shown in Figure 14. After the cotransformation of S_3_-RNase with F-box1 and S_3_-RNase with F-box2, a strong YFP fluorescence signal was detected in the nucleus of onion cells, while YFP fluorescence was not observed in the negative control. Thus, S_3_-RNase interacts with both F-box1 and F-box2(Figure 14).

## 3. Discussion

S-RNase belongs to the RNase T2 family and is the most widely distributed ribonuclease involved in SI. It is present in *Solanaceae*, *Rosaceae*, *Rutaceae* and *Scrophulariaceae*. At present, it is clear that S-RNase, as the pistil determinant of the ‘XiangShui’ lemon SI reaction, is specifically expressed in the style. At the same time, S-RNase exerts its ribonuclease function, which can destroy the pollen tube structure, degrade the pollen tube microfilament cytoskeleton, inhibit pollen tube growth and development and exert a series of toxic effects.

By comparing and analysing the genomic, transcriptomic, ubiquitin-modified-omic and proteomic data of ‘XiangShui’ lemon [12], we screened a total of eight S-RNase genes belonging to the RNase T2 family. According to the ubiquitin-modified-omic dataset, an RNase T2 gene was found accidentally, which was detected in the proteomic data, but not in ubiquitin-modified-omic data. Some proteins that can be degraded by ubiquitination are not detectable among ubiquitin-modified-omic data [14]. It was suggested that the RNase T2 protein is ubiquitinated and degraded during pollination, but whether it is involved in the process of SI needs further verification. In the genome of ‘XiangShui’ lemon, the eight RNase T2 genes were named S_1_-RNase to S_8_-RNase. S_1_-RNase and S_2_-RNase in ‘XiangShui’ lemon have been cloned previously [11,13], so we named the gene S_3_-RNase, which is a newly discovered RNase T2 gene.

The characteristics of the eight RNase T2 gene family members were analysed, and it was found that, except for the low molecular weights of S_5_-RNase and S_6_-RNase (which were only 10.92 and 10.07 kDa, respectively), the molecular weights of other S-RNases ranged from 20–32 kDa. This was slightly different from the traditional S-RNase proteins, whose molecular weights range from 25–32 kDa [15,16], which may be because the individual genes changed throughout their genetic evolution.

Promoter cis-acting element analysis showed that the eight RNase T2 genes contained multiple light-responsive, ABA, IAA, GA, SA and dehydration-responsive elements, suggesting that S-RNases may be involved in a series of self-defence responses, stress responses and hormone responses [17,18]. However, calmodulin-binding sites and phosphate formation response elements were found in the promoter region of S_3_-RNase, indicating that S_3_-RNase likely participates in a series of reactions involving calcium ions (Ca^2+^) and phosphate. Subsequently, this was confirmed by our Y2H experiments.

Through chromosome localisation, we can know that S_8_-RNase is linked to S_7_-RNase, S_4_-RNase is linked to S_2_-RNase, S_1_-RNase is closely linked to S_6_-RNase, S_3_-RNase is distributed on chromosome 4 alone, but S-RNase is not distributed on chromosomes 1, 3, 5, 7 and 8. Therefore, the chromosome distribution of those genes is not uniform.

According to the phylogenetic relationship analysis, eight S-RNases were clustered dispersedly, with the closest relationship with *Citrus clementina* and *Citrus sinensis*, followed by *Oryza sativa subsp* and *Arabidopsis thaliana* and the furthest relationship with *Rosaceae* and *Solanaceae*. However, it is interesting that the S_3_-RNase of ‘XiangShui’ lemon is far from S-RNases of other species, but it can cluster with three S-RNases that have been reported to have self-incompatibility with cherry CaS_4_-RNase, CaS_9_-RNase and CaS_3_-RNase in the same branch, and in the study of cherry, CaS_4_-RNase also interacts with an F-box gene with FBA domain to participate in self-incompatibility, so we are more certain that S-RNase in ‘XiangShui’ lemon may participate in self-incompatibility in the same way [19]. In previous research reports, it is the first condition that the S-RNase gene involved in self-incompatibility has a T2 RNase conserved domain. Therefore, we analysed the conserved domain of 8 S-RNases in ‘XiangShui’ lemon, and the results all meet this prerequisite. Therefore, we can carry out the next screening experiment of S-RNase genes that may participate in self-incompatibility.

Through tissue expression pattern analysis, we found that among the eight RNase T2 genes, only S_1_-RNase, S_2_-RNase, S_3_-RNase and S_4_-RNase were highly expressed in the pistils. Therefore, in follow-up experiments, these four genes were mainly studied. On the basis of the findings of previous studies, we analysed the spatiotemporal expression patterns of S_3_-RNase and S_4_-RNase. The results showed that the expression of S_3_-RNase in stigma increased significantly at 20 h and peaked on the second day. The expression of the S_3_-RNase gene after self-pollination was significantly higher than that after cross-pollination at all times. In the styles, the expression increased significantly at 20 h and peaked on the third day, which was approximately 10 times higher than that at 0 h. In the ovaries, the expression of this gene increased significantly at 20 h and peaked on the second day. The expression after self-pollination was significantly higher than that after cross-pollination at all times. The research results of a previous study showed that both self-pollinated and cross-pollinated pollen could germinate on the stigmas and extend downwards but curled in the lower part of the stigma after 24 h and ultimately could not reach the ovaries [20]. The results of our spatiotemporal expression pattern analysis were consistent with this finding. However, the spatiotemporal expression pattern of the S_4_-RNase gene was not consistent with the pollen growth pattern after pollination, the expression in the stigmas and styles did not change significantly with time, and there was no significant difference in the expression between self-pollination and cross-pollination. Therefore, we speculated that the S_3_-RNase gene may be involved in SI.

To explore the proteins that interact with S-RNases, a yeast cDNA library of ‘XiangShui’ lemon was constructed for Y2H experiments. The experimental results revealed 41 blue monoclonal colonies from S_3_-RNase, and 12 blue monoclonal colonies were obtained from S_4_-RNase. According to the sequencing results, these candidate proteins could be roughly divided into seven categories: (1) hormone regulatory proteins, (2) proteins affecting pollen development, (3) ubiquitin-related proteins, (4) enzymatic proteins, (5) transmembrane transport proteins, (6) stress response-related proteins and (7) mitochondrion-related proteins.

The identified PII, PME51 and PAE8 were pectinase proteins. Previous studies have found that PII and PME were specifically expressed in the pollen, and the high similarity of their sequence with that of the Bp19 of Brassica suggested that they may also be related to pollen development [21]. The components of the pollen tube wall contain pectin [22]. The pectin structure of the pollen tube cell wall can be hydrolysed by S_3_-RNase by interacting with these three zymoproteins, thereby destroying the growth and development of the pollen tube.

APA1 belongs to the aspartic proteinases, which are one of the main proteolytic enzymes. APA1 participates in the PCD of anther tapetum cells and has an important impact on the development of pollen [23]. Aspartic proteases, A36 and A39, were specifically expressed in pollen, playing an important role in the growth and guidance of pollen tubes [24]. We speculated that S_3_-RNase inhibited its activity by interacting with aspartic protease, making it unable to guide pollen tube growth, and the pollen tube growth and development were destroyed. Studies have shown that double mutants with defects in PDAT1 and DGAT1 experience pollen death, and PDAT1 plays a key role in the normal growth of pollen [25]. TIM14-2 is an important component of the transporter complex through which proteins enter mitochondria [26]. In the process of pollen tube cell PCD, mitochondria are regulated by several PCD components (Bcl-2 protein family, Apaf-1). These proteins may be transported to mitochondria through transporters such as TIM14-2 to function, causing changes in mitochondrial membrane potential, mitochondrial swelling and other reactions [27].

RRP46 is a component of the RRP46/CRN-5 homodimer. This homodimer has been identified as a nuclease related to cell death and can perform RNA degradation functions alone. The RRP46/CRN-5 homodimer is involved in the degradation of cellular DNA, causing PCD [28]. In the process of pollen tube growth and development, the degradation of the cell DNA was the main cause of pollen tube PCD in the present study, but it was not clear whether pollen tube DNA degradation was promoted by S-RNase directly. In the process of cell death in animals, Caspase family proteins are involved in the degradation of cell DNA [29], while in plants, PCD only in the process of Papaver SI involves Caspase [30]. This study revealed that RRP46 and APA1 proteins interacting with S-RNase may regulate and participate in the degradation of pollen tube DNA of ‘XiangShui’ lemon, which provides a new avenue for PCD in the process of SI of ‘XiangShui’ lemon.

p24 is a member of a membrane protein family; this protein mainly releases secretory substances into transport vesicles through vesicular transport and is responsible for protein transport in cells [31]. The p24beta2 candidate protein we identified belongs to this family of proteins. We speculated that p24beta1 likely facilitates the transport of S_3_-RNase in pollen tube cells, such that S_3_-RNase can better function in the pollen tubes.

Serine-tRNA ligase, PAP15 and FKGP were simultaneously screened by S_3_-RNase through a Y2H assay, suggesting that the phosphorylation level in pollen tubes was also affected by S_3_-RNase. Studies have found that S-RNase interacts with MdPPa in apple to inhibit MdPPa activity, resulting in PPI accumulation, the aminoacylation of tRNA^Gly^ and tRNA^Ala^ being blocked, protein synthesis being inhibited and pollen tube growth ultimately being inhibited [8]. AtPAP15 in Arabidopsis is specifically expressed in the pollen, hydrolyses inositol phosphate and is the key phosphatase in the process of pollen germination [32,33]. In this study, serine-tRNA ligase, PAP15 and FKGP interacted with S_3_-RNase, indicating that the decomposition of PPI was possibly affected and that the aminoacylation of tRNA^Ser^ or other tRNAs by S_3_-RNase in ‘XiangShui’ lemon involved interactions with FKGP and PAP15 proteins with similar pyrophosphatase activity.

S-RNase interacts strongly with MdCaMBP in *Malus domestica*; as a calmodulin-binding protein, MdCaMBP was shown to be inhibited by S-RNase and could not function as a cyclic nucleotide phosphodiesterase, resulting in cAMP accumulation and affecting the concentration gradient of Ca^2+^ in pollen tubes [10]. From the top to the base of the pollen tube, the calcium concentration gradient from high to low is an important factor for the normal growth of the pollen tube. If the concentration of Ca^2+^ in the pollen tube is affected, the concentration gradient changes, and the polar growth of the pollen tube becomes affected [9]. Therefore, the change in the calcium concentration gradient in the pollen tube can be used as a signal of SI.

The GDT1-like protein 4 screened through our Y2H assays is a calcium transporter located on the cell membrane and may play a role in facilitating the transmembrane transport of Ca^2+^. When the promoter of S_3_-RNase was analysed, two calmodulin-binding sites, both of which were CGCGBOXAT, were found in the promoter, located at -892 (CCGCGT)-897 bp and -850 (CCGCGT)-855 bp. These two results together confirmed that, by transporting and regulating calcium ions, S_3_-RNase likely affects the level of calcium in pollen tubes to achieve SI.

The F-box protein is the pollen determinant of SI S-alleles; F-box proteins are specifically expressed in pollen and are an important component of the SCF complex. In the process of cross-pollination, S-RNase is ubiquitinated, which is marked by the SCF complex and degraded by the 26S proteasome, resulting in the inability of S-RNase to play a series of roles in SI. Previous studies have shown that SFB (F-box), which has an FBA domain, interacts with S-RNase in *Prunus avium* and *Pyrus spp*. [4,19]. However, the S-RNase that interacts with the F-box protein has not been found in the *Rutaceae* family. Fortunately, in the previous experiment, the ubiquitin-modified-omic data and the proteomic data were compared, and one of the S-RNases was detected in the proteomic data but not in the ubiquitin-modified-omic data, which implied that the protein is likely ubiquitinated and degraded through a series processes associated with SI. To understand the S-RNase function, a yeast cDNA library of ‘XiangShui’ lemon was screened, which revealed two F-box genes. Yeast point-to-point assays and BiFC experiments showed that S_3_-RNase interacted with F-box1 and F-box2. Between them, F-box1 has an FBA domain and was specifically expressed in pollen, which is highly consistent with the structural expression of the classic F-box gene of SI. Therefore, we speculated that the F-box gene is most likely a component of the SCF complex that constitutes the ubiquitination marker of S-RNase. It was confirmed that S_3_-RNase was involved in the process of SI.

**Figure 15:**
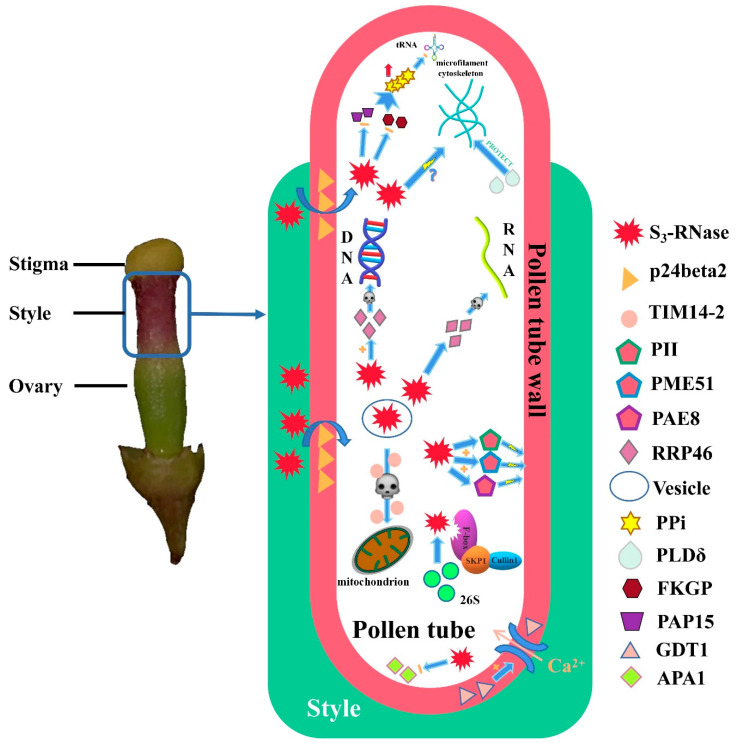
Putative regulatory network of S_3_-RNase in SI.

Based on the interactions involving S-RNases, an SI pattern diagram centred on the S_3_-RNase of ‘XiangShui’ lemon was preliminarily constructed (Figure 15). All protein functions were listed in Supplementary Table S3. However, the regulatory mechanism of each part remains unclear. Therefore, our future research will focus on the regulatory mechanism of the SI interaction network centred on S-RNase.

## 4. Materials and Methods

### 4.1. Plant Materials

The flowers of 8-year-old ‘XiangShui’ lemon (*Citrus × limon ‘Rosso’*) trees in the Fruit Trees Specimen Garden of the Agricultural College of Guangxi University were emasculated, self-pollinated (‘XiangShui’ lemon♀ × ‘XiangShui’ lemon♂) or cross-pollinated (‘XiangShui’ lemon♀ × ‘BaiHua’ lemon♂) and bagged. Stigma, style and ovary samples of ‘XiangShui’ lemon were collected from five ‘XiangShui’ lemon trees with similar growth at 0 h, 10 h, 20 h, 1 d, 2 d, 3 d and 4 d after self-pollination or hybridisation. Tissue samples (leaves, stigmas, styles, ovaries, anthers, filaments, petals, receptacles) were collected from the same five trees. Tissue samples: collect at least three ‘XiangShui’ lemon flowers from each of the five trees. Spatiotemporal samples: pollinate at least five flowers of each of the five trees at each time period, and then collect these flowers. The collected samples were immediately put into a −80 °C freezer for preservation.

### 4.2. Cloning and Bioinformatics Analysis of Four S-RNase Genes

A HiPure HP Plant RNA MiNi Kit RNA Extraction Kit (Guangzhou Magen Biotechnology Co., Ltd., Guangzhou, China) was used to extract the total RNA of ‘XiangShui’ lemon, which was then stored at −80 °C. cDNA was synthesised according to the instructions of a reverse transcription kit (Takara Bio, Kusatsu, Japan) used.

Primers for cloning were designed according to eight S-RNase gene sequences retrieved from ‘XiangShui’ lemon ubiquitin-modified-omic, transcriptomic and whole genomic data (unpublished) [34]. The specific primers S_3_-RNaseF, S_4_-RNaseF and S_3_-RNaseR, S_4_-RNaseR were designed. PCR amplification was carried out with cDNA used as a template.

The identification of S-RNase nucleotide sequences was performed through the National Center for Biotechnology Information (NCBI) BLAST program (http://www.ncbi.nlm.nih.gov/BLAST, accessed on 2 May 2022).

BioXM 2.6 software (Ji Huang, Nanjing, China) was used to translate nucleotide sequences into amino acid sequences. Homologous amino acid sequences of other species were obtained via BLAST of the NCBI, DNAMAN 6.0 (Lynnon Biosoft, Vaudreuil-Dorion, Quebec, Canada) was used for sequence alignment and MEGA 11 (Koichiro Tamura, Hachioji, Tokyo, Japan) was used to construct an evolutionary tree. NCBI CD-Search was used for conservative domain predictions (https://www.ncbi.nlm.nih.gov/cdd) (accessed on 7 July 2022), which revealed the function of S-RNase interacting proteins via the UniProt website (https://beta.uniprot.org/, accessed on 7 July 2022) [35], ExPASy was used to predict the physical and chemical properties of the proteins (https://web.expasy.org/protparam/, accessed on 7 July 2022) [36] and IBS was used to form a nucleotide structure map [37].

After the cis-acting elements of the S_3_-RNase and S_4_-RNase promoter regions were analysed and predicted by the PLACE (https://www.dna.affrc.go.jp/PLACE/?action=newplace) (accessed on 7 July 2022) and PlantCARE (http://bioinformatics.psb.ugent.be/webtools/plantcare/html/) (accessed on 7 July 2022) online databases, TBtools was used for the visualisation of the results [38]. Regulatory elements were identified by analysis involving the PlantCARE database and PLACE database. SOPMA (https://npsa-prabi.ibcp.fr/cgi-bin/npsa_automat.pl?page=npsa_sopma.html, accessed on 7 July 2022) [39]. SWISS-MODEL (https://swissmodel.expasy.org, accessed on 7 July 2022) was used to predict protein secondary structures and model the structure of homologue proteins, thus yielding three-dimensional structural models [40].

### 4.3. Quantitative PCR (qPCR) Analysis

We aimed to determine the spatiotemporal expression of the S-RNase genes in different pistil tissues at different times after the self-pollination and cross-pollination of ‘XiangShui’ lemon and to determine the tissue expression levels in different flower tissues of ‘XiangShui’ lemon.

qPCR primers were designed using Primer Premier 3.0, and the ClActin gene was used as the thane internal control [41]. All the primers used are listed in Supplementary Table S1. cDNA amplification was performed as described by Wang [42]. Each of the samples included three technical replicates and was analysed using the 2^−∆∆CT^ method [43].

### 4.4. Subcellular Localisation

Agrobacterium tumefaciens EHA105 containing pBI221-S_1_-RNase-EGFP, pBI221-S_2_-RNase-EGFP, pBI221-S_3_-RNase-EGFP and pBI221-S_4_-RNase-EGFP (Wuhan GeneCreate Biological Engineering Co., Ltd., Wuhan, China) fusion vector or the pBI221-EGFP control vector were subsequently infiltrated into onion (*Allium cepa*) [44]. The pBI221-EGFP vector and the target gene fragment were digested by *BamHI*/*SalI*. The enzyme digestion system was 50 μL, gene segment was 20 μg, 10 × cutsmart buffer 5 μL. Double endonuclease (one for each) μL. Make up deionised water to 50 μL, 37 °C, 1–2 h. A TIANGEN DNA purification and recovery kit is used to obtain purified enzymatic products. Refer to the kit instructions for the steps. The purified target fragment was incubated in 1 U T4 DNA Ligase and ligated with plasmid at 16 °C for 1 h to construct recombinant plasmid. Briefly, 4′,6-Diamidino-2-phenylindole (DAPI) was subsequently used to stain the nuclei. Images at a wavelength of Ex/Em = 488/507 nm were captured with a confocal microscope (TCS-SP8MP; Leica, Wetzlar, Germany).

### 4.5. Construction of a Y2H Library

Mixed samples of ‘XiangShui’ lemon flower organ tissues such as style, filament, pollen, leaf, petal, stigma, ovary and receptacle tissues were collected and ground thoroughly. The same two copies of total RNA were extracted with a Takara MiniBEST Plant RNA Extraction Kit (TaKaRa Code No. 9769). Using the SMART cDNA Library Construction Kit (Clontech Code No.634901) and Advantage 2 PCR Kit (Clontech Code No.639206), 3 µL of total RNA was used to synthesise cDNA. The purified cDNA was normalised using a TRIMMER DIRECT cDNA Normalisation Kit (Evrogen Cat#NK003). The processed cDNA was amplified via PCR using a cDNA Normalisation Kit and an Advantage 2 PCR Kit to obtain normalised cDNA. The amplified cDNA was purified with a TaKaRa MiniBEST DNA Fragment Purification Kit (TaKaRa Code No.9761) and then dissolved in dH2O. The cDNA was digested by SfiI restriction enzymes. CHROMA SPIN-1000-TE (Clontech Code No.636079) was used to remove short fragments from the digested cDNA. After PCI/CI purification, the cDNA was refined with ethanol and dissolved in dH2O. Using a DNA ligation Kit, the pGADT7-SfiI three-frame vector was ligated to an appropriate amount of cDNA at 12 °C via O/N Connect. The obtained linker was purified to obtain a primary cDNA library.

A small amount of primary library linker fragments was taken and electrotransformed into competent HST08 cells. A proper amount of transformed bacterial solution was applied to Amp-resistant LB plates and cultured overnight at 37 °C. The primary library capacity was calculated via the number of colonies growing on the plate, and the inserted fragments were detected by 1.5% agarose gel electrophoresis. According to the library capacity test data, the volume of the required connecting solution was calculated, electrotransformed into the competent HST08 cells, applied to large Luria-Bertani (LB) plates, and cultured overnight at 37 °C. The amplified colony was recovered, and the plasmid was extracted with a NucleoBond Xtra Midi EF Kit (MN Code No. U0420B) to obtain the amplified library plasmid.

The library plasmid was subsequently transformed into Y187 competent cells (refer to Y187 competent cell transformation instructions for specific operation instructions), coated onto plates (the media of which excluded Leu) and incubated at 30 °C for 72 h. The yeast on the plate was collected with 2× YPDA, and the final concentration of 25% glycerol was maintained at −80 °C for subsequent experiments.

### 4.6. Y2H Assays

pGBKT7-S_1_-RNase, pGBKT7-S_2_-RNase, pGBKT7-S_3_-RNase and pGBKT7-S_4_-RNase fusion vectors were constructed to serve as bait sequences and transferred into Y2H Gold competent cells to obtain bait yeast. The pGBKT7 vector and the target gene fragment were digested by *NdeI*/*EcoRI*, the enzyme digestion system was 50 μL and the gene segment was 20 μg, 10 × cutsmart buffer 5 μL. Double endonuclease (one for each) μL. Make up deionised water to 50 μL, 37 °C, 1–2 h. A TIANGEN DNA purification and recovery kit is used to obtain purified enzymatic products. Refer to the kit instructions for the steps. The purified target fragment was incubated in 1 U T4 DNA Ligase and ligated with plasmid at 16 °C for 1 h to construct recombinant plasmid. Self-activation verification was performed. The library screening experiment was conducted according to the Matchmaker^TM^ Gold Yeast Two-Hybrid System User Manual (TaKaRa). The screened genes were subjected to yeast colony PCR and sequencing analysis. The plasmid of the prey protein was extracted and transformed into DH5α competent cells, after which the transformed bacteria were incubated on LB media (containing corresponding antibiotics) at 37 °C for 24 h and subjected to Escherichia coli colony PCR. The Escherichia coli plasmid (pGADT7-prey) was then extracted and transferred into Y187 competent cells. pGBKT7-T was used to detect the self-activation of the prey proteins. A point-to-point assay was performed to verify the interaction between nonself-activated prey proteins and S-RNases.

### 4.7. BiFC Assays

Full-length S-RNase and prey proteins were cloned into puc-sPYNE or puc-sPYCE and transformed into GV3101 competent cells. The activated target colony was inoculated into LB liquid culture media (containing corresponding antibiotics), shaken overnight at 28 °C, mixed for 24 h at the same volume and centrifuged (8000 rpm for 5 min) at 4 °C. The supernatant was discarded, and MS liquid media (containing acetosyringone and MgCl_2_) diluted to an OD600 of 0.7~1.0 were added. The fresh onion epidermis was placed into the mixture to allow infection for 20–30 min. The onion epidermis was subsequently removed and spread onto MS solid medium. After 16 h of light culture, the fluorescence was observed under a confocal scanning microscope (TCS-SP8MP, Leica, Germany).

## Figures and Tables

**Table 1:** Characteristics of 8 S-RNase gene family members in ‘XiangShui’ lemon.

Gene Name	Gene Entry Number	Chromosome	Chromosomal Position	Length (aa)	pI	MW (kDa)
*S_1_-RNase*	ON981301	Chr9	29,869,547–29,908,462 +	177	10.60	20.40
*S_2_-RNase*	ON981301	Chr6	1,799,000–1,799,808 +	235	9.52	27.11
*S_3_-RNase*	ON981303	Chr4	14,622,505–14,626,122 +	278	5.89	31.47
*S_4_-RNase*	ON981304	Chr6	310,580–311,366 +	222	8.98	25.74
*S_5_-RNase*	ON981305	Chr9	19,297,972–19,298,225 −	98	7.95	10.92
*S_6_-RNase*	ON981306	Chr9	29,869,583–29,869,942 +	89	10.18	10.07
*S_7_-RNase*	ON981307	Chr2	4,443,631–4,444,675 −	230	5.20	25.82
*S8-RNase*	ON981307	Chr2	4,440,309–4,441,515 +	194	4.93	25.76

## Data Availability

The data presented in this study are available on request from the corresponding author.

## Supplementary Materials

The following supporting information can be downloaded at: https://www.mdpi.com/article/10.3390/ijms231810431/s1.
