# Supplementary material for: Genome-Wide Analysis of the RNase T2 Family and Identification of Interacting Proteins of Four ClS-RNase Genes in ‘XiangShui’ Lemon
Source: Int J Mol Sci. 2022 Sep 9;23(18):10431. doi: 10.3390/ijms231810431 (PMC9499183; doi:10.3390/ijms231810431)
Supplement: Supplementary file 1 [file ijms-23-10431-s001.zip › Supplementary Tables S1-S3.pdf]

Supplementary Table S1 PCR primer list.

Primer	Primer sequence (5′-3′)	Annotation
S_1-RNase	F: ATGCATTTTAGTATTGTCAA; R: TCATTGTGGGATTCGCATCA	PCR
S_2-RNase	F: ATGAAGGTTAACCTCTTATT; R: CTAGTCGTTACGATTAGCCG	PCR
S_3-RNase	F: CTGATTCGACTATCCCTCGT; R: GGCATTATAGTCAGGCCACAGT	Gene expression
S_4-RNase	F: GGCCATCAGATTTCGTTCTA; R: GTCTGATAGCTGCTTGGAGA	Gene expression
S_5-RNase	F: CAGACGGTGGCAGTTACAAT; R: GGGAGGAACGTCAAACATGA	Gene expression
S_6-RNase	F: GTTTCTTGCATCGCATTCGC; R: GGTATTTGTCGAGGACCTGC	Gene expression
S_7-RNase	F: CAGCGGCAGATTTCGGGATT; R: GCACAGACTCAGAGCAAGTG	Gene expression
S_8-RNase	F: GATTTTGGCATTCACGGGCT; R: CAGACTCTGCACAAGTACCGT	Gene expression
F-box1	F: GATAAGGGGAAGAATGCAAC; R: TTACGGAAGGAATAAGACTG	Gene expression
F-box2	F: GATTTCCTGATGGGTTGCCT; R: TGCACATCCGAACAACATCC	Gene expression
ClActin	F: CGTATGAGCAAGGAAATCACAG ; R: ATTGATCCTCCAATCCAAACAC	

Supplementary Table S2 Secondary structure.

	Alpha helix	Beta sheet	Turn	Coil
S_1-RNase	33.33%	17.51%	5.08%	44.07%
S_2-RNase	31.91%	11.91%	4.68%	51.49%
S_3-RNase	42.09%	9.71%	3.24%	44.96%
S_4-RNase	34.23%	14.86%	5.86%	45.05%
S_5-RNase	29.59%	19.39%	12.24%	38.78%
S_6-RNase	18.18%	30.68%	5.68%	45.45%
S_7-RNase	36.52%	12.17%	6.09%	45.22%
S_8-RNase	34.51%	13.72%	5.31%	46.46%

Supplementary Table S3 protein functions.

S$_3$-RNase Interaction candidate protein information		
Number	Gene description	Molecular function
**A1**	Pectin acetylesterase 8 (PAE8)	Pectin hydrolase activity.
**A2**	Polygalacturonase inhibitor (PGIP1)	It is an important factor for plant resistance to phytopathogenic fungi.
**A3**	Putative invertase inhibitor (PPI)	Acts in the modification of cell walls via demethylesterification of cell wall pectin, Related to pollen development.
**A4**	Transmembrane emp24 domain-containing protein p24beta2-like (p24beta2)	Involved in vesicular protein trafficking.
**A5**	Serine-tRNA ligase	Serine-tRNA ligase activity, seryl-tRNA aminoacylation.
**A6**	Purple acid phosphatase 15 (PAP15)	Affect pollen germination, with acid phosphatase activity.
**A7**	F-box/kelch-repeat protein At1g15670(F-box2)	Component of SCF(ASK-cullin-F-box) E3 ubiquitin ligase complexes, which may mediate the ubiquitination and subsequent proteasomal degradation of target proteins.
**A8**	F-box protein At5g03970-like(F-box1)	Component of SCF(ASK-cullin-F-box) E3 ubiquitin ligase complexes, which may mediate the ubiquitination and subsequent proteasomal degradation of target proteins.
**A9**	Bifunctional fucokinase/fucose pyrophosphorylase(FKGP)	Pyrophosphorylase activity.
**A10**	Aspartic proteinase A1(APA1)	Possesses aspartic protease activity, played an important role in the growth and guidance of pollen tubes.
**A11**	Exosome complex exonuclease RRP46 homolog(RRP46)	Possesses hydrolytic DNase and phosphorolytic RNase activities in vitro.
**A12**	Probable pectinesterase/pectinesterase inhibitor 51(PME51)	Acts in the modification of cell walls via demethylesterification of cell wall pectin.
**A13**	Mitochondrial import inner membrane translocase subunit TIM14-2	An important component of the transporter complex for proteins to enter mitochondria.
**A14**	Phospholipid:diacylglycerol acyltransferase 1	The Double-mutant defect of PDAT1 and DGAT1 lead to pollen death, PDAT1 played a key role in the normal growth of pollen.
**A15**	Gibberellin receptor GID1B	Functions as soluble gibberellin (GA) receptor. Seems to be required for GA signaling that controls flower development.
**A16**	GDT1-like protein 4	Calcium ion transmembrane transport.

S_4-RNase Interaction candidate protein information		
Number	Gene description	Molecular function
B1	serine/threonine-protein phosphatase PP1 isozyme 4(TOPP4)	Acts as positive regulator in the gibberellin (GA) signaling pathway to regulate plant growth and development.
